# A versatile T cell-based assay to assess therapeutic antigen-specific PD-1-targeted approaches

**DOI:** 10.18632/oncotarget.25591

**Published:** 2018-06-12

**Authors:** Maarten Versteven, Johan M.J. Van den Bergh, Katrijn Broos, Fumihiro Fujiki, Diana Campillo-Davo, Hans De Reu, Soyoko Morimoto, Quentin Lecocq, Marleen Keyaerts, Zwi Berneman, Haruo Sugiyama, Viggo F.I. Van Tendeloo, Karine Breckpot, Eva Lion

**Affiliations:** ^1^ Laboratory of Experimental Hematology, Vaccine and Infectious Disease Institute (VAXINFECTIO), Faculty of Medicine and Health Sciences, University of Antwerp, Antwerp, Belgium; ^2^ Laboratory for Molecular and Cellular Therapy, Vrije Universiteit Brussel, Brussels, Belgium; ^3^ Department of Cancer Immunology, Osaka University Graduate School of Medicine, Osaka, Japan; ^4^ Department of Cancer Immunotherapy, Osaka University Graduate School of Medicine, Osaka, Japan; ^5^ *In Vivo* Cellular and Molecular Imaging Laboratory, Vrije Universiteit Brussel, Brussels, Belgium; ^6^ Nuclear Medicine Department, UZ Brussel, Brussels, Belgium; ^7^ Division of Hematology, University Hospital Antwerp, Antwerp, Belgium; ^8^ Center for Cell Therapy and Regenerative Medicine, University Hospital Antwerp, Antwerp, Belgium

**Keywords:** PD-1/PD-L1 immune checkpoint pathway, antigen-specific, bioassay, flow cytometry, immune checkpoint inhibition

## Abstract

Blockade of programmed cell death protein 1 (PD-1) immune checkpoint receptor signaling is an established standard treatment for many types of cancer and indications are expanding. Successful clinical trials using monoclonal antibodies targeting PD-1 signaling have boosted preclinical research, encouraging development of novel therapeutics. Standardized assays to evaluate their bioactivity, however, remain restricted. The robust bioassays available all lack antigen-specificity. Here, we developed an antigen-specific, short-term and high-throughput T cell assay with versatile readout possibilities. A genetically modified T cell receptor (TCR)-deficient T cell line was stably transduced with PD-1. Transfection with messenger RNA encoding a TCR of interest and subsequent overnight stimulation with antigen-presenting cells, results in eGFP-positive and granzyme B-producing T cells for single cell or bulk analysis. Control antigen-presenting cells induced reproducible high antigen-specific eGFP and granzyme B expression. Upon PD-1 interaction, ligand-positive antigen-presenting immune or tumor cells elicited significantly lower eGFP and granzyme B expression, which could be restored by anti-PD-(L)1 blocking antibodies. This convenient cell-based assay shows a valuable tool for translational and clinical research on antigen-specific checkpoint-targeted therapy approaches.

## INTRODUCTION

The importance of the immune checkpoint receptor programmed cell death protein 1 (PD-1) and its ligands programmed cell death ligand 1 (PD-L1) and programmed cell death ligand 2 (PD-L2) in the regulation of immune responses has gained vast scientific interest in the past decade. While this pathway is physiologically indispensable in controlling auto-reactive T cells, it has been demonstrated that tumors and infectious diseases can exploit this pathway to suppress the immune system and promote immune escape in favor of their persistence [1, 2]. Interaction of PD-1 expressed on T cells with its ligands results in immune suppression through interference with the physiological T-cell receptor (TCR) signaling pathway [3]. From meta-analyses, we now know that overexpression of surface or soluble PD-L1 in multiple tumor types is mostly associated with a poor prognosis [4–6]. Providentially, tumor cell PD-L1 expression has been put forward in different malignancies as a predictive biomarker for higher responsiveness to anti-PD-(L)1 therapy [7–9]. Recent clinical trials, for example in melanoma, have demonstrated a favorable clinical outcome after anti-PD-1 treatment in patients with high PD-L1 expression [10]. These findings have led to large pharmaceutical investments in the investigation and development of antibodies blocking this pathway, directed to either the receptor or its ligands [11–13]. Success of this approach is illustrated by the growing list of clinical trials implementing anti-PD-(L)1 antibodies (over 500 registered clinical trials) in more than 200 different malignancies on clinicaltrials.gov (April 26th, 2018) and has recently resulted in US FDA and EMA approvals of the anti-PD-1 antibodies nivolumab (Bristol-Myers Squibb, US) and pembrolizumab (Merck, US) and the anti-PD-L1 antibodies atezolizumab (Genentech, US), avelumab (Merck & Pfizer, US) and durvalumab (Medimmune/AstraZeneca, US).

Evaluation of the bioactivity of PD-1 or PD-L1 blocking antibodies, was performed by a number of research groups [14–17]. Although their research offers reliable characterization of such antibodies, long-term and laborious experimental protocols and the need for primary cells makes its usage complicated to standardize. The growing need of bioassays to test anti-PD-1/PD-L biologicals like antibodies or other targeted moieties [18], led to the development and marketing of PD-1/PD-L1 blockade bioassays by a selected number of companies. All current commercially available assays (Amsbio, UK; BPS Bioscience, USA; DiscoverX, USA; Explicyte, France; GenScript, China; Promega, USA) employ bioluminescent luciferase as a nuclear factor of activated T cells (NFAT)-driven reporter gene in the commonly used genetically modified Jurkat T cell line to measure polyclonal TCR-mediated effector T-cell activation. Under normal circumstances, TCR triggering leads to activation of NFAT-proteins and subsequent transcriptional regulation of downstream genes which bear an NFAT-response element (NFAT-RE) in their promoter region [19]. Binding of PD-1 with its ligands disturbs the TCR signaling, resulting in debilitated NFAT activation and downstream NFAT-regulated gene expression [3, 20, 21]. As stimulator, these assays optionally provide stably engineered PD-L1-expressing artificial antigen-presenting cells (aAPCs) to activate the Jurkat cells in an antigen-independent manner. Quantification of TCR activation in the absence versus presence of a PD-1/PD-L-targeted molecule is assessed based on luciferase activity. The high-throughput potential (mostly 96-well plate automated readout) and short-term assay duration (1-day) makes these off-the-shelf bioassays highly valuable in compound screening.

With the rise of cellular immunotherapy approaches, PD-1-targeted (combination) therapies have gained particular interest [22]. These approaches aim at promoting antigen-specific tumor targeting by the immune system while overcoming immune evasion and suppression by the tumor micro-environment [23]. Preclinical data provide evidence of increased T cell activation after combined cellular therapy with PD-1 pathway blockade [24–26] and a number of clinical trials evaluating the synergistic effect of anti-PD-(L)1 and cellular therapy like dendritic cell (DC) therapy (e.g. NCT01067287, www.clinicaltrials.gov) or adoptive T-cell transfer (NCT02621021), are ongoing. Since none of the currently available PD-1-targeted bioassays provide antigen-specific or multiparametric information, we further expanded the possibilities of these assays by developing a new cellular antigen-specific PD-1-sensitive bioassay with varied readout possibilities. By engineering a genetically modified TCR-deficient Jurkat T cell line, we developed a versatile PD-1-sensitive, multiparametric T cell assay with antigen-specific properties combined with short-term assay duration and high-throughput potential comparable with the already available bioassays. Customizable antigen-specificity through transfection of messenger RNA (mRNA) encoding for the TCR of interest, enables this assay to be employed in various fields of research. This assay not only allows screening of PD-1/PD-L-targeted compounds, but as well allows evaluation of antigen-specific immunogenicity of therapeutics that indirectly target the PD-1 pathway like some cellular therapies (e.g. dendritic cell vaccines [12]. In this context, our assay could accelerate studies on antigen-specific checkpoint-targeted cell interactions and therapies, providing a valuable tool for both translational and clinical research on PD-1-targeted cellular immunotherapy strategies.

## RESULTS AND DISCUSSION

### Generating off-the-shelf PD-1^+^ antigen-specific T cell lines

**Figure 1:**
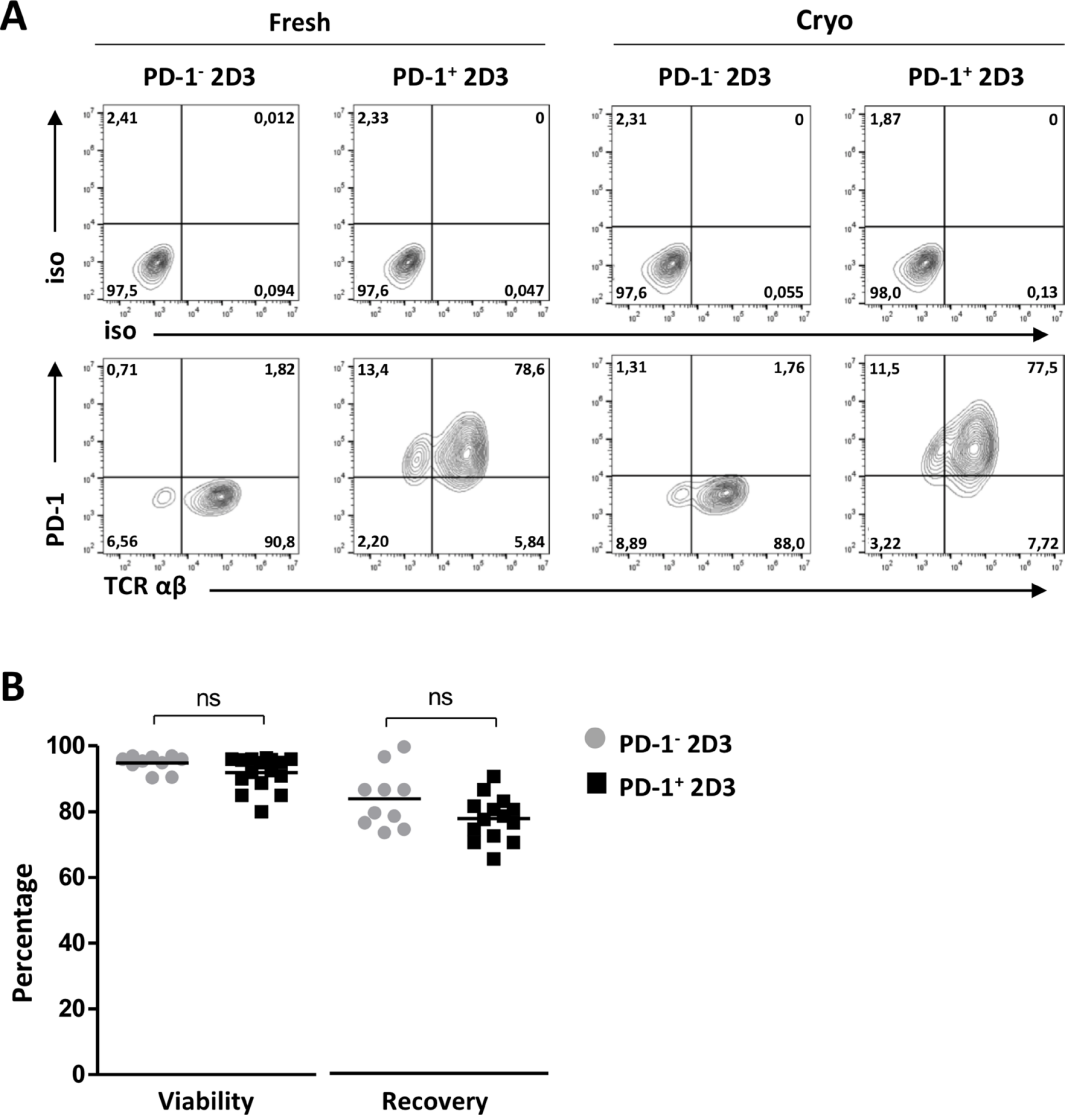
Efficiency of PD-1 transduction, *TCR* mRNA electroporation and cryopreservation of 2D3 cells (**A**) Representative flow cytometry T-cell receptor (TCRαβ) and programmed death-1 (PD-1) protein surface expression profiles and corresponding isotype controls of non-transduced PD-1^−^ 2D3 and PD-1-transduced (PD-1^+^) 2D3 cells 24 hours after *TCR* mRNA electroporation (fresh; 10-14 replicates) and after thawing of *TCR* mRNA-electroporated cells (cryo; 6 replicates). (**B**) Percentage viability and recovery upon *TCR* mRNA electroporation of PD-1^−^ and PD-1^+^ 2D3 cells. Data information: in (B), means are depicted. ^*^P ≤ 0.05 (Student’s *t*-test). Abbreviations: cryo, transfected effector cells were cryopreserved prior to co-culture; fresh, stimulator cells were co-cultured immediately following transfection; ns, not significant; PD-1, programmed death-1 protein; TCR, T-cell receptor.

To obtain a readily available PD-1-positive (PD-1^+^) T cell model for assessment of PD-1/PD-L axis involvement in T-cell signaling, the PD-1-negative 2D3 (PD-1^−^ 2D3) cell line – a derivative of the established Jurkat T cell line characterized by CD8 expression, lack of endogenous TCR and expression of enhanced Green Fluorescent Protein (eGFP) under the control of NFAT promoter – was stably transduced to express surface PD-1 (PD-1^+^ 2D3; Figure 1). The absence of an endogenous TCR allows for reliable introduction of a TCR of interest. Both 2D3 non-transduced and PD-1-transduced 2D3 cells could reproducibly and efficiently be transfected with Wilms’ Tumor-1 (WT1) or glycoprotein 100 (gp100) epitope-specific *TCR* mRNA, using our in-house developed mRNA electroporation method [27–29], resulting in high levels of transgene TCRαβ surface expression 24 hours after transfection of PD-1^−^ 2D3 (89.3 ± 2.1%) as well as of PD-1^+^ 2D3 (89.3 ± 1.5%) cells (Figure 1A, Fresh). Control mock-electroporated PD-1^−^ and PD-1^+^ 2D3 cells remained completely negative for TCRαβ. Viability of both PD-1^−^ (94.8 ± 0.8%) and PD-1^+^ 2D3 cells (91.9 ± 1.2%) remained high 24 hours after transfection and respectively 83.3 ± 2.8% and 77.3 ± 1.8% cells could be consistently recovered (Figure 1B). Evaluating its off-the-shelf use, TCR-positive 2D3 cells were aliquoted for cryopreservation and were assessed for viability and stability of PD-1 and TCRαβ surface expression after thawing. As illustrated in the quadrant plots both PD-1 and TCRαβ (87.0 ± 4.3% for PD-1^−^ 2D3, 88.3 ± 1.4% for PD-1^+^ 2D3 cells) expression remained stable (Figure 1A, Cryo). Thawed PD-1^−^ and PD-1^+^ 2D3 cells were viably recovered (97.7 ± 0.6% and 97.0 ± 0.7%, respectively). Furthermore, these amenable PD-1^−^ or PD-1^+^ 2D3 cell lines are easy to maintain in regular culture medium and are not subjected to any enrichment and cytokine-supplemented expansion protocols unlike primary or transduced antigen-specific T cell clones which are laborious and often difficult to maintain in culture [30]. With mRNA electroporation, highly pure TCR-positive T cells can be readily generated, swiftly adaptable to the antigen under investigation, facilitating the development of a variety of PD-1-sensitive antigen-specific T cell models. Our optimized electroporation procedure results in stable expression up to at least 72 hours after electroporation [29]. However, when preferred, stable transduction with a TCR of interest could further simplify the assay protocol to better mimic primary antigen-specific T cell clones, while precluding repetitive mRNA transfections.

### Introduced TCR triggers robust antigen-specific T-cell activation

**Figure 2:**
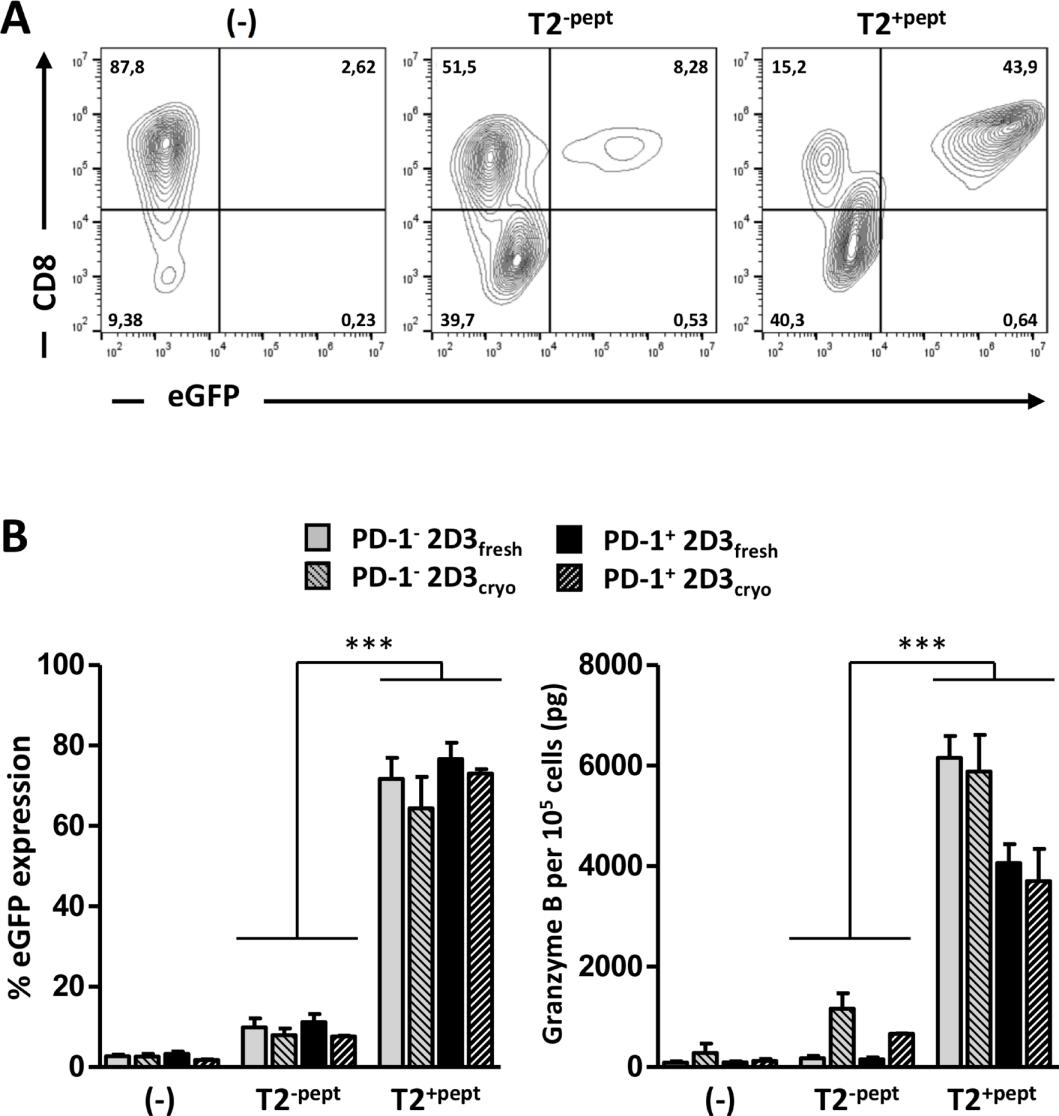
Validation of antigen-specific TCR function of transfected 2D3 and PD-1^+^ 2D3 cells (**A**–**B**) Activation profiles of freshly used or thawed WT1-specific *TCR* mRNA-electroporated PD-1^−^ and PD-1^+^ 2D3 cells left unstimulated (-) versus 24 hours co-culture with unloaded (T2^-pept^) and WT1 peptide-pulsed (T2^+pept^) stimulator cells at a ratio of 2:1. Comparable results were obtained with gp100 TCR-positive PD-1^−^ and PD-1^+^ 2D3 cells. (A) Representative example of TCR activation-mediated eGFP expression within the viable CD8^+^ cell population as assessed with flow cytometry (freshly used *WT1 TCR* mRNA-electroporated PD-1^+^ 2D3 cells). (B) The left graph shows the mean percentage (± SEM) WT1-specific TCR activation-mediated eGFP expression from 2–8 replicate experiments. The right panel depicts the mean amount (± SEM) of secreted granzyme B determined with ELISA in cell-free 24-hour culture supernatant of 10^5^ effector cells for 2–4 replicate experiments. Data information: ^*^P ≤ 0.05, ^**^P < 0.01, ^***^P < 0.001 (one-way ANOVA). Abbreviations: cryo, transfected effector cells were cryopreserved prior to co-culture; eGFP, enhanced green fluorescent protein; fresh, stimulator cells were co-cultured immediately following transfection; PD-1, programmed death-1 protein; SEM, standard error of mean; TCR, T-cell receptor; WT1, Wilms’ tumor 1.

To validate that high TCR surface expression corresponds with antigen-specific functionality, *TCR* mRNA-electroporated PD-1^−^ or PD-1^+^ 2D3 cells were stimulated with the prototypic antigen-presenting T2 cell line, which is negative for PD-L expression and thus serves as a PD-1-independent assay control (Figure 2). With the eGFP gene under control of a promoter containing an NFAT-RE, TCR-signaling can be measured without the need for substrate addition and enzymatic conversion. Direct expression of green fluorescence enables a variety of live-cell assaying; from highly sensitive single-cell multiparametric flow cytometry and sorting of activated T cells for downstream analyses up to real-time *in vitro* (e.g. IncuCyte^®^) and *in vivo* [31, 32] live-cell imaging. Applying conventional multiparametric flow cytometry, co-cultures of 2D3 cells with T2 cells were stained for CD8 surface expression to discriminate effector cells from stimulator cells. After selection of viable CD8^+^ T-cells, percentage of eGFP positivity distinctly reflected the magnitude of activation (Figure 2A). In the two different TCRαβ models (WT1 and gp100) tested, stimulation with relevant peptide-loaded T2 cells (T2^pept+^) proved reproducibly equal antigen-specificity and response magnitude of PD-1^−^ 2D3 and PD-1^+^ 2D3 cells with mean ranges of eGFP positivity of [64.4–76.6%] and [74.5–88.2%] for the WT1 and gp100 models, respectively (*P* < 0.001 for all T2^pept+^ versus T2^pept-^ conditions). T2 cells on their own (T2^pept-^) elicited low non-specific levels of eGFP (<11.2% for WT1, <14.2% for gp100) comparable to previously described T2-mediated background responses [33, 34], not significantly different from unstimulated (-) effector cells with a background of < 5% eGFP expression (Figure 2B, left graph). Comparable data were generated by two independent laboratories for both model antigens, showing low inter- and intra-assay variability, emphasizing assay reproducibility and translatability.

Complementary to single-cell analysis, cell-free supernatant of 24-hour co-cultures was collected. As an alternative accessible low-cost readout, analysis of granzyme B secretion of T2 co-cultures with replicate *TCR* mRNA electroporations of PD-1^−^ or PD-1^+^ 2D3 cells was performed at the same time with ELISA (Figure 2B, right graph). Background granzyme B levels of TCR-positive PD-1^−^ 2D3 and PD-1^+^ 2D3 cell monocultures (-) reached 92.9 ± 21.2 pg/10^5^ cells and 97.9 ± 20.7 pg/10^5^ WT1 TCR^+^ T cells (Figure 2B, right graph) and 62.6 ± 2.5 pg/10^5^ and 61.1 ± 1.2 pg/10^5^ gp100 TCR^+^ T cells, respectively. Non-specific granzyme B secretion (T2^pept-^), like non-specific eGFP expression, remained at background levels for all TCR-positive T cells (not significant for all T2^pept-^ versus unstimulated (-) conditions). In line with the flow cytometry data, antigen-specific (T2^pept+^) TCR-mediated activation of PD-1^−^ 2D3 and PD-1^+^ 2D3 cells was reflected by significantly higher granzyme B levels, comparable for both WT1 and gp100 T cell models, reaching mean concentrations of 6155.8 ± 376.1 pg/10^5^ for PD-1^−^ and 4063.6 ± 320.7 pg/10^5^ for PD-1^+^ WT1 TCR^+^ 2D3 cells (Figure 2B, right graph; *P* < 0.001) and 5187.6 ± 740.6 pg/10^5^ for PD-1^−^ and 3856.6 ± 703.2 pg/10^5^ for PD-1^+^ gp100 TCR^+^ 2D3 cells. Not affecting eGFP signaling, a significantly positive but lower granzyme B secretion by the PD-1-transduced 2D3 cells is observed of which the exact mechanism remains to be elucidated. Elaborating on flow cytometric analysis, granzyme B production could also be detected following intracellular staining, enabling simultaneous analysis of TCR activation and cytotoxicity-related granzyme B production at the single-cell level. This opens the way to expand the combination of surface and intracellular activation markers of interest. Valuing the effect of cryopreservation on the functionality of *TCR* mRNA-electroporated T cells, functional experiments were repeated pairwise with thawed transfected T cells (cryo) and compared with T cells immediately after transfection (fresh) (Figure 2B). Both multiparametric single-cell (eGFP) and bulk surrogate cytotoxicity (granzyme B) readouts showed equivalent antigen-specific T-cell activation by fresh and cryopreserved TCR^+^ 2D3 cells without significant loss of functionality. The immediate use of thawed TCR-specific effector T cells for downstream analyses, without the need to expand, culture or further engineer the effector cells endorses the potential of this versatile T cell model assay for off-the-shelf use.

### TCR^+^ PD-1^+^ 2D3 cells as a tool to assess involvement of PD-1-signaling in antigen-specific immunotherapy approaches

**Figure 3:**
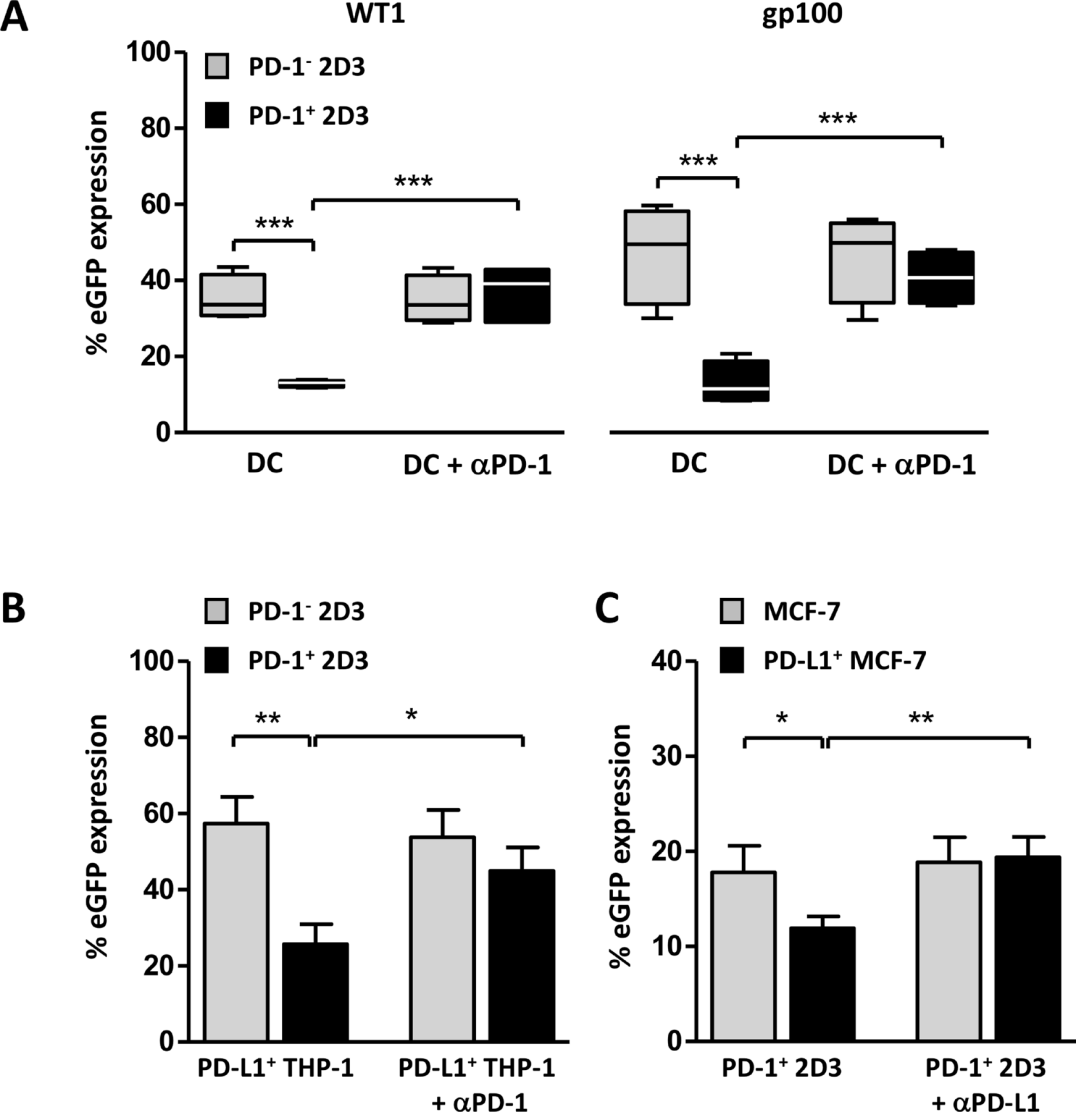
TCR^+^PD-1^+^ 2D3 cells as a model assay for evaluation of involvement of PD-1 signaling in cell-mediated antigen-specific T-cell activation (**A**–**C**) WT1 (A, B) and gp100 (A, C) specific T-cell activation expressed as percentage viable CD8^+^ eGFP^+^ PD-1^−^ and PD-1^+^ 2D3 cells (± SEM) after 24 hours co-culture with different PD-L1^+^ stimulator cells. Neutralizing antibody against PD-1 (αPD-1; A, B; 15 µg/mL in WT1 model (A, B), 5 µg/mL in gp100 model (A)) or PD-L1 (αPD-L1; C) was added to cells 1 hour prior to co-culture to verify PD-1-mediated signaling, where indicated. (A) PD-1-dependent stimulating capacity of two differently generated peptide-pulsed mature monocyte-derived dendritic cells (WT1 (4 DC donors tested in two independent experiments); gp100 (4 DC donors in four independent experiments)). (B)Impact of induced PD-L1 expression on peptide-pulsed THP-1 leukemic cells on WT1-specific T-cell activation (6 replicate experiments). (C) gp100-specific PD-1^+^ T cell-activating capacity of peptide-pulsed wild-type or stably transduced PD-L1^+^ MCF-7 breast carcinoma cells (4 replicate experiments). Data information: in A, the horizontal line represents the median percentage eGFP expression (*n* = 4). ^*^P ≤ 0.05, ^**^P < 0.01, ^***^P < 0.001 (repeated measures one-way ANOVA with Bonferroni post-hoc test). Abbreviations: eGFP, enhanced green fluorescent protein; gp100, glycoprotein 100; PD-1, programmed death 1 protein; PD-L1, programmed death-ligand 1; TCR, T-cell receptor; WT1, Wilms’ tumor 1.

The currently available bioluminescent cell-based PD-1/PD-L1 blockade bioassays provide a good option for rapid screening of therapeutic antibodies or other compounds developed to interfere with PD-1-signaling interaction. Artificial PD-L1^+^ stimulator cells and a T cell activator are mostly provided, sufficient to measure potency and stability of antibodies. Given the generic TCR activation, these assays lack the opportunity to evaluate defined antigen-targeted cellular immunotherapies. We aimed to fill this gap with the TCR^+^ PD-1^+^ 2D3 bioassay, to evaluate tumor antigen-targeted monocyte-derived DC vaccines, known to express significant levels of PD-L1 and PD-L2 [35, 36]. In the absence of PD-1, specific stimulation of TCR^+^ 2D3 cells with peptide-pulsed DCs resulted in significant WT1-specific (35.3 ± 3.0%) and gp100-specific (47.2 ± 6.5%) eGFP expression (mean ± SEM, *n =* 4; Figure 3A). Activation of PD-1^+^ 2D3 cells with antigen-targeted DCs resulted in significantly lower eGFP expression for both WT1 (12.7 ± 0.5%) and gp100 (12.8 ± 2.8%) T cell models, corresponding with a 64% and 73% lower signal as compared to the PD-1^−^ 2D3 cells. Validating the mechanism of action, blocking PD-1-transduced 2D3 cells with anti-PD-1 antibody (pembrolizumab) could fully rescue T cell activation to the levels of PD-1^−^ 2D3 cells (eGFP expression of 36.0 ± 4.0% for WT1 and 40.7 ± 3.6% for gp100), while there was no effect of the antibody on non-transduced 2D3 cells. PD-L surface expression on DCs has been demonstrated to suppress T cell activity in various malignancies [37, 38] and blockade was shown to enhance T cell functions [39]. In the context of DC-based immunotherapy, it has been suggested that in addition to providing immunostimulatory signals [40–42] the immunopotency of DC vaccination could be improved by suppressing inhibitory checkpoint pathways [12, 35, 43, 44]. With this versatile bioassay, reproduced in two prototypic tumor antigen models, antigen-specific T cell activating capacity of PD-1-targeted approaches in combination with DC-based therapy can be robustly assessed.

In the search for valuable prognostic and predictive biomarkers, PD-L1 tumor expression has drawn the attention [5, 8]. Ambiguous when it comes to prognosis, its predictive value remains debatable [8]. In this context, the applicability of the immunologic PD-1^+^ 2D3 assay for assessment of antigen-specific PD-L1^+^ tumor-mediated immune suppression and the effect of checkpoint blockade was evaluated. PD-L1^+^ THP-1 leukemic stimulator cells, consistently generated upon 24-hour recombinant human (rh) interferon-gamma (IFN-γ) stimulation (86.5 ± 6.7% PD-L1^+^ after 24-hour IFN-γ stimulation, 6 replicates), served as WT1-specific stimulator cells for co-culture with WT1 TCR-positive PD-1^−^ or PD-1^+^ 2D3 cells (Figure 3B). In the absence of PD-1, peptide-pulsed PD-L1^+^ tumor cells induced high levels of eGFP (57.4 ± 7.0%; mean ± SEM of 6 replicate experiments), while PD-1-triggering resulted in significantly lower eGFP expression (25.7 ± 5.3%). Confirming involvement of PD-1-signaling, the suppressed eGFP response could be significantly recovered by blocking PD-1 with antibody (45.0 ± 6.1%), but not to levels of PD1^−^ 2D3 cells (53.8 ± 7.2%). Unlike the full recovery by the same anti-PD-1 antibody when PD-1^+^ 2D3 cells are stimulated with DCs (Figure 3A), even double antibody concentrations could not strengthen the PD-L1^+^ THP-1-induced response. At the level of the stimulator cells, PD-L1 expression is comparably high for IFN-γ-stimulated THP-1 cells (mean ± SEM; 86.5 ± 6.7% PD-L1^+^) and monocyte-derived dendritic cells (moDC) (94.5 ± 1.1% PD-L1^+^, *n =* 4), while its other ligand PD-L2 is not on the surface of IFN-γ-stimulated THP-1 cells and significantly expressed by moDC (51.2 ± 8.9% PD-L2^+^, *n =* 4). Suggesting that other signals could contribute to the inhibitory PD-1-signaling pathway, the exact mechanism remains to be elucidated. The gp100 TCR^+^ PD-1^+^ 2D3 model assay was tested with MCF-7 breast carcinoma cells that were stably transduced with PD-L1 (> 75% PD-L1^+^). In analogy with the PD-L1^+^ leukemic cells, stimulation with PD-L1^+^ peptide-loaded MCF-7 cells resulted in significantly lower eGFP expression (11.9 ± 1.2%) as compared to their non-transduced MCF-7 counterparts (17.8 ± 2.8%; mean ± SEM of 4 replicate experiments, Figure 3C). Abrogating PD-1 signaling with PD-L1 blocking antibody could completely overcome this tumor-mediated T cell suppression (19.4 ± 2.2%) to the levels of eGFP expression in the absence of PD-L1 (19.0 ± 2.6%), highlighting the relevance of PD-1/PD-L1 checkpoint blockade as an immunotherapeutic modality.

### Conclusion and future perspectives

In the development process of antigen-specific immune therapy approaches targeting the inhibitory PD-1/PD-L pathway, we encountered a lack of availability of robust and user-friendly T-cell bioassays. Therefore, we sought to develop a versatile cellular antigen-specific PD-1-sensitive T-cell assay with comprehensive readout possibilities. The here presented assay is unique because of (i) its straightforward customizable antigen-specificity, (ii) its short-term assaying, (iii) its high-throughput potential and (iv) its readily accessible nature. It enables robust research on combinatorial antigen-specific PD-1-targeted immune therapies. Beyond the PD-1/PD-L1 pathway, a collection of additional immune checkpoints are being assessed, including T cell immunoglobulin- and mucin-containing molecule 3 (TIM-3), lymphocyte activation gene 3 protein (LAG-3) and T cell immunoglobulin and ITIM domain (TIGIT) [45]. Looking ahead, the concept of this versatile 2D3 cellular assay could be applied for any immune checkpoint or other targeted molecules of interest, paving the way for swift screening of pathway involvement and of new antigen-specific targeted therapeutics. In this context, a comparable jurkat-based reporter system designed to evaluate activating co-signals rather than checkpoint inhibitors was recently published [46], confirming the need for robust bioassays for evaluation of TCR-targeted therapy approaches. With the advance feature of stimulating with primary antigen-presenting cells, rather than merely artificial antigen-presenting moieties decorated with a known ligand of the immune checkpoint receptor under investigation, this cellular bioassay permits research on immune checkpoint receptors for which the binding partner(s) is unknown. Hence, antigen-specific TCR immune checkpoint receptor-engineered 2D3 cells could provide a valuable tool to accelerate emerging translational and clinical research on antigen-specific checkpoint-targeted cell interactions and therapy approaches.

## MATERIALS AND METHODS

### Ethics statement and primary cell material

This study was approved by the Ethics Committees of the University Hospital Antwerp/ University of Antwerp (Antwerp, Belgium) and of the Brussels University Hospital/Free University of Brussels (Brussels, Belgium) under the reference numbers 16/35/357 and 2013/198, respectively. Experiments were performed using blood samples from anonymous human leukocyte antigen (HLA)-A^*^0201-positive donors provided by the Blood Service of the Flemish Red Cross (Mechelen, Belgium) and Blood Transfusion Center of the University Hospital Brussel (Brussels, Belgium).

### Effector cells

2D3 cells were generated from TCR-deficient Jurkat 76 cells (human acute T cell leukemia) by transduction with a *CD8 alpha-E2A-CD8 beta* construct (both Jurkat 76 cells and CD8-encoding plasmid were kind gifts of Prof. Hans Stauss, Institute of Immunity and Transplantation, University College London, London, UK) and with a plasmid vector containing the enhanced green fluorescein protein (*eGFP*) gene under the control of a nuclear factor of activated T-cell (NFAT)-dependent promoter (NFAT-eGFP plasmid [47] was kindly provided by Prof. Takashi Saito, Riken Research Center for Allergy and Immunology, Yokohama, Japan). PD-1^+^ 2D3 cells were generated by transduction of the 2D3 cell line with a human PD-1 plasmid [48], at a multiplicity of infection (MOI) of 5, using the protocol described previously to transduce human dendritic cells [49]. The production of lentiviral vectors and their characterization by flow cytometry was performed as previously described in Goyvaerts *et al.* [50]. Both PD-1^−^ and PD1^+^ 2D3 cells were maintained in Roswell Park Memorial Institute 1640 (RPMI) culture medium (Gibco Invitrogen) supplemented with 10% fetal bovine serum (FBS; Gibco Invitrogen).

### Stimulator cells

The human lymphoblastic HLA-A^*^0201^+^-restricted TAP-deficient T2 cell line (kindly provided by Dr Pierre Van der Bruggen; Ludwig Institute for Cancer Research, Brussels, Belgium), routinely used to study T-cell activation, was maintained in Iscove’s Modified Dulbecco’s Medium (IMDM; Gibco Invitrogen) supplemented with 10% FBS. The human leukemic HLA-A^*^0201^+^ THP-1 cell line (ATCC) was stimulated for 24 hours with 40 ng/mL rhIFN-γ) to induce PD-L1 protein expression and maintained in RPMI culture medium (Gibco Invitrogen) with 10% FBS (Gibco Invitrogen). The HLA-A^*^0201^+^ MCF-7 breast carcinoma cell line (ATCC) was stably transduced with a plasmid encoding human PD-L1 [48] at a MOI of 10 according to the previously described protocol (*vide supra*) and maintained in RPMI medium supplemented with 10% FBS, 2 mmol/L L-glutamine (Sigma-Aldrich), 100 U/mL penicillin, 100 µg/mL streptomycin (Sigma-Aldrich), 1 mmol/L sodium pyruvate and non-essential amino acids (Sigma-Aldrich). All cell lines were maintained in logarithmic growth phase at 37° C in a humidified atmosphere supplemented with 5% CO_2_. Peripheral blood mononuclear cell (PBMC) isolation and subsequent monocyte isolation for moDC generation was performed as previously described in Van den Bergh *et al.* [35] or Tuyaerts *et al.* [51], where indicated.

### mRNA and transfection of PD-1^−^ 2D3 and PD-1^+^ 2D3 cells

The human WT1_37–45_-specific TCR gene was generated as previously described [52]. The human gp100_280–288_ TCRα and TCRβ vectors were kindly provided by Prof. Niels Schaft (Research group leader of the RNA-group, Department of Dermatology, Universitätsklinikum Erlangen, Erlangen, German) [53]. mRNA transcripts were generated using an mMessage mMachine T7 *in vitro* transcription kit (Life Technologies) according to the manufacturer’s protocol. *WT1 TCR* mRNA (1 µg/10^6^ cells) or *gp100 TCR*α and *TCR*β mRNA (up to 2,5 µg of each per 10^6^ cells) was transfected in PD-1^−^ 2D3 or PD-1^+^ 2D3 cells in 200 µL Opti-MEM reduced serum medium without phenol red (Life Technologies) in a 4 mm electroporation cuvette (Cell Projects) using Square Wave settings (500 V, 5 ms, 0 gap, 1 pulse; *WT1 TCR* mRNA) or a time constant protocol (300 V, 7 ms; *gp100 TCR* mRNA) of a Gene Pulser Xcell^™^ device (Bio-Rad Laboratories). *TCR* mRNA-electroporated cells were used for co-culture 2–4 hours after transfection or aliquoted in FBS + 10% dimethyl sulfoxide (DMSO) as cryopreservation medium for controlled freezing at −80° C. Viability (flow cytometry, *vide infra*) and cell count (automated ABX Micros 60 cell counter; Horiba) were measured after electroporation and after thawing in pre-warmed IMDM + 10% FBS. The percentage recovery was calculated by dividing the cell count post electroporation with the cell count prior to electroporation, multiplied by 100.

### Co-cultures

*TCR* mRNA-electroporated PD-1^−^ 2D3 or PD-1^+^ 2D3 cells were cultured in triplicate in a 96-well round-bottom plate in IMDM + 10% FBS. Where indicated, cells were preincubated for 1 hour with 5–15 µg/mL anti-PD-1 antibody (Pembrolizumab, Keytruda; Merck Sharp & Dohme Limited) or 1 µg/200 µL neutralizing anti-PD-L1 antibody (clone MIH1; eBiosciences, cat. no. 16-5983-82) prior to stimulation. Stimulator cells (*vide supra*), either unloaded or pulsed with 10 µg/mL WT1_37–45_ (VLDFAPPGA; JPT) or with 50 µg/mL gp100_280–288_ (YLEPGPVTA; Eurogentec) peptide, were added to PD-1^−^ 2D3 or PD-1^+^ 2D3 effector cells at optimized effector-stimulator ratios of 2:1 (WT1-specific experiments) or 10:1 (gp100-specific experiments) and co-cultured for 24 hours at 37° C, 5% CO_2_ (unless specified otherwise).

### Flow cytometry analysis

Viability of *TCR* mRNA-transfected PD-1^−^ 2D3 and PD-1^+^ 2D3 cells was assessed by propidium iodide (PI) staining immediately after transfection and at least 1 hour after thawing of cryopreserved cells. *TCR* mRNA electroporation efficiency was evaluated 24 hours after transfection (pre- and post- cryopreservation) using a phycoerythrin (PE)-labeled anti-human TCRαβ monoclonal antibody (Miltenyi Biotec). Twenty-four hours after stimulation, PD-1^−^ and PD-1^+^ 2D3 TCR activation-mediated eGFP expression was determined on cell pellets after surface staining with anti-human PE or allophycocyanin (APC)-H7-conjugated CD8 (BD), followed by 10-minute incubation with 7-aminoactinomycin D (7-AAD; BD) to distinguish between viable and dead cells. All flow cytometric acquisitions were performed on CytoFLEX (Beckman Coulter) or Fortessa (BD) instruments.

### Granzyme B ELISA

Secretion of the cytotoxin granzyme B by PD-1^−^ and PD-1^+^ 2D3 cells was determined in 24-hour co-culture supernatant with an enzyme-linked immunosorbent assay (ELISA; R&D) following the manufacturer’s instructions and acquired on a Victor 3 multilabel counter (Perkin Elmer). A sample dilution of 1:8 was optimal to fit the standard curve of the kit with a detection limit of 2.4 pg/mL and top standard of 833.3 pg/mL.

### Statistical analysis

Flow cytometry data were analyzed using FlowJo software (v10.2, TreeStar Inc). Prism software (v5, GraphPad) was used for graphing and statistical calculations. Data were analyzed using (repeated measures) one-way analysis of variance (ANOVA) followed by Bonferroni’s post-hoc test. Results were considered statistically significant when *P*-value was less than 0.05.
